# Varied Age of First Presentation of Sickle Cell Disease: Case Presentations and Review

**DOI:** 10.1155/2021/8895020

**Published:** 2021-02-08

**Authors:** Alexis Claeys, Susanne Van Steijn, Lydia Van Kesteren, Elizabet Damen, Machiel Van Den Akker

**Affiliations:** ^1^Pediatric Department, ZNA Paola Children's Hospital, Antwerp, Belgium; ^2^Pediatric Department, UZ Gent, Gent, Belgium; ^3^Pediatric Hematology, Oncology Unit, UZ Brussel, Jette, Belgium; ^4^Pediatric Hematology, Oncology Unit, Queen Mathilde Mother and Child Center, Antwerp University Hospital, Edegem, Belgium

## Abstract

Sickle cell disease is a multisystem condition characterized by hemolytic anemia and vasoocclusion. Not only are the symptoms of the first presentation but also the ages of presentation are very variable. Following three case reports, different causes of possible late presentation are discussed. Many factors are responsible for the age at which sickle cell disease is diagnosed: doctor's delay (unfamiliarity with the disease), patient's delay (education and financial position of the parents, cultural factors), high- versus low-resource country (availability of newborn screening), fetal hemoglobin, reticulocyte count, and genetic modulators, such as SCD genotype, alpha-thalassemia, fetal hemoglobin concentration, and G6PD deficiency. The individual course of sickle cell disease depends on (epi) genetic and environmental properties and the underlying interactions. In further studies, the role of each factor should be evaluated more deeply, and its use as a marker of disease severity or activity should be assessed.

## 1. Introduction

Sickle cell disease (SCD) is a common group of life-threatening, genetic disorders caused by the synthesis of abnormal hemoglobin (sickle hemoglobin), which when deoxygenated, polymerizes and causes sickling of red blood cells. SCD is characterized by chronic hemolytic anemia, vasoocclusion, and progressive vascular injury causing multiorgan damage [1]. The clinic of SCD is quite variable and reflects the interactions with other genetic and environmental factors and can include painful vasoocclusive crises (VOCs), acute chest syndrome, cerebral stroke, and acute and chronic hemolysis. Due to functional hyposplenism, there is susceptibility to invasive bacterial infection with encapsulated bacteria [2, 3].

In sub-Saharan Africa, the Middle East, India, and the Mediterranean area, SCD is the most prevalent, where more than 90% of the annual SCD births take place, but due to migration, SCD can be diagnosed also in other parts of the world [4]. While newborn screening and follow-up with prophylactic interventions are common in high-resource countries, most children die before the age of 5 years, often before a diagnosis was made, in low-resource countries [5].

Sickle cell anemia (SCA) refers solely to patients homozygous for the sickle mutation (HbSS) and is together with sickle *β*°-thalassemia responsible for more severe diseases in the group of SCD. Hemoglobin SC disease and sickle *β*⁺-thalassemia are common and in general, less severe presentations of SCD. Hemoglobin SD and hemoglobin SE are much less common.

The individual course of SCD is heterogeneous and depends not only on several genetic factors, such as the concomitant presence of alpha-thalassemia, sickle *β*-haplotypes, fetal hemoglobin levels, and G6PD (glucose-6-phosphate dehydrogenase) deficiency, but also on environmental properties and their underlying interactions [2]. There can be a widespread diversity in clinic and first presentation between different communities, but sometimes also in the same family [6–8]. A late first presentation is not always so straightforward, and patient and doctor's delay is not uncommon as we will show in two cases.

## 2. Case Presentations

### 2.1. Case 1

A twenty-month-old boy with no relevant medical history presented at the outpatient clinic with limping for 7 days (*D*7 = day 7 of disease) without a prior trauma. Fever up to 38.5 degrees Celsius was noted over the last week. The boy was born at term by an uncomplicated vaginal delivery following a normal pregnancy; his weight at birth was 3.6 kilograms. He was the first child of healthy, nonconsanguineous parents from Angola. The family history did not reveal any relevant information except that the mother is a carrier of sickle cell anemia (HbAS), which was denied to the father. Physical examination showed a mild sick, male with normal cardiovascular and respiratory parameters, with 38.5 degrees Celsius. The skin was pale, without signs of jaundice. There were no signs of dehydration. ENT examination showed mild rhinorrhea with some pharyngeal redness. Heart and lung auscultation was normal. There was no hepatosplenomegaly. Neurological examination was normal. We saw mild limping of the right leg with mildly painful abduction right hip. The initial complete blood count showed a mild, microcytic anemia with hemoglobin of 9.4 g/dL (normal 10.5–13.6 g/dL), MCV 68 (normal 70–85 fL), reticulocytes 60/1000 RBC (normal 11–29/1000 RBC), leukocytes of 12.3 × 10E9/L (normal 6.0–17.0 × 10E9/L) with normal differentiation, and thrombocytes of 559 × 10E9/L (normal 304–574 × 10E9/L). Blood smear revealed poikilocytosis, tear cells, schistocytes, and some target cells. C-reactive protein (CRP) was elevated up to 144 mg/L (normal ≤ 5 mg/L). An ultrasound of the ankles, knees, and hips did not show any abnormalities. Under suspicion of reactive arthritis, the patient was sent home with bedrest, pain medication, and follow-up appointment.

At D13, he was again seen in the outpatient clinic; his temperature normalized over the last few days, while his physical examination did not reveal changes. The ultrasound of the hip was normal. A radiograph of the pelvis showed irregularities of the right iliac wing. Bone scintigraphy with 99mTc-MDP revealed an elevated distribution at the right pelvic wing anterolaterally, possible of an inflammatory nature. Because CRP spontaneously improved (23.9 mg/L (normal ≤ 5 mg/L)) and of the absence of fever, an MRI of the pelvis was requested as an outpatient.

At D19, MRI (magnetic resonance imaging) revealed an increased signal of the right iliac wing on the STIR (short-TI inversion recovery) images. The adjacent soft tissue captures the contrast. The image suggests osteomyelitis. The patient was admitted. CRP normalized to 3 mg/l. His temperature was normal. After blood cultures were drawn, IV (intravenously) antibiotics, cefotaxime and flucloxacillin, were started. Blood culture did not reveal any growth. Because of an atypical course, more extensive research into possible explanation has been carried out, including cultures for *Salmonella typhi* and *Kingella kingae*, the tuberculin skin test, and serology for HIV. They were all negative. Before a biopsy of the bone lesion was performed, hemoglobin electrophoresis confirmed sickle cell anemia (HbA2 2.3%, HbF 24.5%, and HbS 73.2%). Due to the absence of clear sickle cells in the blood smear, denial of carrier status (HbAS) of the father, late recognition that the limping is pain due to VOC, and the presence of a high HbF, the diagnose of sickle cell anemia was delayed.

### 2.2. Case 2

A three-year-old boy was followed by a pediatric rheumatologist on suspicion of systemic onset juvenile idiopathic arthritis due to varying pains in his hands and feet for 18 months. Sporadically, the child refused to walk. Often, these episodes were preceded by fever and eased with ibuprofen. No rash was reported. He presented in the clinic of the pediatric rheumatologist with fever (39 degrees Celsius) for 1-2 weeks with limping and painful swelling of the right tibia above the ankle. He was initially seen by the pediatric rheumatologist who made the diagnosis of systemic onset juvenile idiopathic arthritis (JIA), and treatment with ibuprofen and corticosteroids was started. The body temperature normalized, but pain and swelling of the tibia remained, after which the child was admitted.

The boy was born at term by an uncomplicated vaginal delivery following a normal pregnancy; his weight at birth was 3 kilograms. He was the first child of healthy, nonconsanguineous parents from Guinea. The family history did not reveal any relevant information.

Physical examination at the first presentation showed a moderate sick male with normal cardiovascular and respiratory parameters, with a temperature of 37.6 degrees Celsius. The skin was pale. No signs of dehydration. No signs of upper respiratory tract infection. Heart and lung auscultation were normal.

The abdomen was showing mild hepatomegaly (3-4 cm under the costal arch), and the spleen was not palpable. Extremities: at the right tibia, warm, painful swelling several centimeters proximal of the distal metaphysis. The left tibia was painful on palpation, but no other clinical signs.

The initial complete blood count showed a severe, normocytic anemia with hemoglobin of 6.4 g/dL (normal 11.5–13.5 g/dL), reticulocytes 77/1000 RBC (normal 8–20/1000 RBC), leukocytes of 15.3 × 10E9/L (normal 5.5–15.5 × 10E9/L) with normal differentiation, and thrombocytes of 448 × 10E9/L (normal 228–433 × 10E9/L). Blood smear revealed hypochromasia, target cells, poikilocytosis, and sickle cells. There were no signs of hemolysis with normal lactate dehydrogenase (707 U/L (normal 425–975 U/L)) and total bilirubin (0.7 mg/dL (normal 0.2–1.3)). C-reactive protein was 56 mg/L (normal ≤ 5 mg/L).

After admission to the pediatric department, within hours, his clinical condition worsened with prolonged capillary refill with tachycardia (192/minute) but normal blood pressure. After blood cultures were drawn, IV ceftriaxone (100 mg/kg) was started together with IV fluid resuscitation (NaCl 0.9%, 20 mL/kg) and red blood cell (RBC) transfusion. X-ray of the right tibia showed significant soft tissue swelling from the distal lower leg and at the distal metaphysis, a slight clearance with possible cortex interruption. Bone scintigraphy with 99mTc-MDP reveals a bone injury at the right distal tibia with a hyperemic component, compatible with an abscess. Surgical drainage showed a lot of pus with a culture showing growth of *Salmonella typhimurium*. Antibiotic treatment was switched to high dose amoxicillin according to the sensitivity. Blood and stool cultures remained negative. Hemoglobin electrophoresis confirmed the diagnosis of sickle cell disease (HbA2 2.7%, HbF 24.1%, and HbS 73%). The initial wrong diagnosis (doctor's delays) by not recognizing sickle cell disease despite high risk based on ethnicity and VOC and the high HbF were responsible for the late diagnosis.

### 2.3. Case 3

A three-year-old girl without a relevant medical history was admitted to the hospital under the suspicion of pneumonia. She was born at term by an uncomplicated vaginal delivery following a normal pregnancy; her weight at birth was 3.2 kilogram. She was the second child of healthy, nonconsanguineous parents from Mali. The family history did not reveal any relevant information. Physical examination showed a sick female with mild tachycardia (pulse 134/minute) and moderate dyspnea (34/minute), with 39.1 degrees Celsius. The skin was pale, no signs of jaundice, and no signs of dehydration. ENT examination showed the presence of mild rhinorrhea with some pharyngeal redness. Systolic murmur 1/6 was over the heart and diminished air-entry over the right lower lobe. The abdomen was not showing hepatosplenomegaly. The initial complete blood count showed a mild, microcytic anemia with hemoglobin of 8.5 g/dL (normal 10.5–13.6 g/dL), reticulocytes of 30/1000 RBC (normal 11–29/1000 RBC), leukocytes of 10.3 × 10E9/L (normal 6.0–17.0 × 10E9/L) with normal differentiation, and thrombocytes of 316 × 10E9/L (normal 229–435 × 10E9/L). Blood smear revealed poikilocytosis, polychromasia, and target cells. C-reactive protein (CRP) was only mildly elevated up to 30 mg/L (normal ≤ 5 mg/L). X-thorax showed a consolidation in the region of the right lower lobe. Under suspicion of pneumonia, intravenous amoxicillin-clavulanic acid was initiated. Despite the ethnicity and the clinic of an acute chest syndrome, only in the context of the anemia, a hemoglobin electrophoresis was performed, revealing the presence of hemoglobin C (42%) and hemoglobin S (46%). Family research showed that the parents are carriers (mother HbC 32% and father HbS 42%), and their first-born male has the same condition as his sister, namely, hemoglobin SC disease. After discharge, antibiotic prophylaxis and folic acid were initiated.

## 3. Discussion

The patients presented in this case series are born in Belgium. They are of sub-Saharan African immigrant families and demonstrate a high frequency of SCD mutations. In their native countries, newborn screening and comprehensive healthcare only rarely takes place at an early age [9], therefore, resulting in a wide variation of the age at diagnosis of SCD. The medium age reported varies from 2 until 6 years [10–12]. Sarat et al. report that in Brazil, the medium age at diagnosis of the adults receiving care for HbSS was five years and for HSC, 21 years [13]. Even in high-resource countries such as Belgium, universal newborn screening and prenatal counseling are not always performed. According to the German SCD registry, most children were discovered after the age of one year, usually by presenting with symptoms [14, 15]. The median age at diagnosis of a cohort of children with SCD in the Netherlands was 25 months [16]. Often due to the lack of knowledge of the physicians regarding SCD, even symptoms, typically associated with clinical manifestations of SCD, are misinterpreted, resulting in inadequate treatment. It is important to address these important public health issues.

There can be several other factors also responsible for the manner and the time at which the disease presents itself, some of which will be discussed (Table 1).

### 3.1. Western World versus Developing Countries

Although patients with SCA have an identical monogenic condition, its clinical phenotypes are highly variable [17, 18]. Different pathways play a role in the pathophysiology, and different genes have been under evolutionary pressure. It is fair to assume that ethnic differences influence the course of SCA [17]. The vast majority and burden of disease is in sub-Saharan Africa. Because of economic limitations, newborn screening of hemoglobin disorders is not achieved. Diagnosis is mostly delayed until children have clinical signs. Screening because of a positive family history is possible yet less applied because of financial considerations [19]. Also, not infrequently, the belief in evil spirits may be one obstacle for seeking timely medical care [20].

In sub-Saharan countries, without intervention, 50–90% of children with SCD will die in childhood, mostly due to infection/sepsis [2, 21], often before the diagnosis of SCD is made. There is no increased mortality in children with severe malaria when they also have sickle cell anemia [22].

Worldwide, we also have a genetic explanation for different ways of presentation, as we know there are different haplotypes in different regions of the world. For example, in the Kingdom of Saudi Arabia, there are two major clinical phenotypes. Patients from the Western Province have a severe course consistent with the Benin haplotype. They are prone to recurrent episodes of acute chest syndrome (ACS), stroke, and dactylitis and in general have a lower level of hemoglobin and hemoglobin F (HbF). They typically present early with painful crises. Patients from the Eastern Province have a milder course of disease; they act more like the Arab and Indian phenotypes. This is associated with higher levels of HbF and frequently a later date of the first presentation [23, 24].

### 3.2. Fetal Hemoglobin

HbF is a physiologic protein tetramer that is of major importance during fetal life. Its affinity for oxygen is higher than the mother's hemoglobin and thereby facilitates the transfer of oxygen from maternal to fetal blood [25]. A higher HbF level in the postnatal period is known to have a positive effect on the course of SCD [2, 25–27]. Patients with SCD and hereditary persistence of fetal hemoglobin (HPFH) have a significantly milder phenotype [27] with a longer lifespan of the red blood cells [18]. HbF prevents polymerization of HbS by reducing the concentration of HbS. The sufficient amount of HbF to prevent the clinical manifestations of SCD in vivo is not completely clarified [27], while in vitro studies suggest that 20% HbF is necessary to prevent polymerization of HbS [28]. In a cross-sectional single center study in Nigeria, it was found that stroke was related to lower levels of HbF; while on the other hand, complications such as priapism, osteomyelitis, and septic arthritis or ulcers were not linked to lower levels of HbF. In this study, lower levels were mostly found in boys, which were not seen in adults. Possibly, a hormonal role during puberty is responsible. In addition, there is a relationship between the percentage of HbF and age, in which the percentage of HbF decreases with age [26].

A different explanation might be the haplotype of the disease. Senegal, Saudi, and Indian haplotypes are generally associated with higher levels of HbF and therefore known for a milder course [24–26]. HbF is also increased in different inherited conditions, such as HPFH, hereditary spherocytosis, and thalassemia. Family studies suggest an inheritance that does not follow the Mendelian pattern. HbF levels are multifactorial, influenced with 89% of variability because of genetic factors, and the other is dependent on sex, age, and environmental factors. The level of HbF rises in several acquired states, such as pregnancy, aplastic anemia, thyrotoxicosis, hepatoma, myeloproliferative disorders, or hypoplastic myelodysplastic syndrome [25].

### 3.3. Reticulocytes

The role of the reticulocyte in the pathophysiology of SCD is not fully understood and still understudied [18]. The reticulocyte count is commonly used as a marker of the bone marrow's response to hemolysis and anemia [18, 27]. Under normal conditions, each second, the bone marrow produces about 2 million reticulocytes. In SCD, this can increase up to 20-fold due to hemolytic anemia. During this increased production, the bone marrow releases reticulocytes prematurely in the blood in order to meet the demands. As SCD is accompanied by chronic hemolysis, there is a continuous stress erythropoiesis and therefore a persistent reticulocytosis. These prematurely released reticulocytes are less rigid than the sickle cell, but they contribute to vasoocclusion due to their big size and membrane properties promoting adhesion [18]. The splenic dysfunction in SCD is responsible for the lack of removal of the reticulocytes, leading to an overrepresentation (up to 25%) of the reticulocytes of all circulating red blood cells. The benefit of these cells might not transcend the pathological consequences. Data and studies are lacking to identify a thorough link between reticulocytosis and disease severity [18].

In infancy, in children with SCD, the physiologic anemia will show an exaggerated increase in the reticulocyte count and could be a marker for SCD. In the cooperative study of sickle cell disease, the absolute reticulocyte count above 200 000/*µ*l in the age group 2–6 months was associated with a three-time risk for SCD-related events which needed hospitalization within the first three years of life [18, 29, 30]. Ambrose et al. showed in a prospective cohort study conducted in Tanzania that children with SCA and sickle *β*°-thalassemia had a lower Hb with a high red cell distribution width (RDW) at 3- and 6-month follow-ups, compared to carriers of sickle cell disease and sickle *β*^+^-thalassemia. The results of a simple blood test could suspect the presence of SCD already in this age group [19].

### 3.4. Genetic Modulators

Some genetic modulators of SCD severity are well known as concurrent *α*-thalassemia, fetal hemoglobin concentration, and *ß*-globin haplotype. Possible G6PD deficiency can have an impact on SCD. More recently, BCL11 A and HBS1L-MYP were added to the list [27].

### 3.5. SCD Genotype

While sickle cell anemia (HbSS) and sickle *β*^0^-thalassemia (HbS-*β*^0^) are responsible for more severe diseases; hemoglobin SC disease (HbSC) and sickle *β*⁺-thalassemia (HbS-*β*⁺) are common and in general, less severe presentations of SCD. HbSC is the most common type in the African American population in the United States [27]. HbC does not polymerize, but it potentiates the polymerization of HbS by increasing the mean corpuscular hemoglobin concentration. As the course is rather mild, the patients still can suffer from the classic vasoocclusive complications as priapism, splenic sequestration, and acute chest syndrome. HbSC patients rarely present with a stroke as they have higher hemoglobin levels [27], but presumable, due to the higher blood viscosity, retinopathy is seen more often [31]. HbS-*β*⁺ is milder with less severe pain crises and less severe splenic dysfunction. Therefore, the disease can present itself later in life [32]. On the other hand, this genotype has a broad spectrum of phenotypes, and therefore, caution should be taken [27].

### 3.6. Alpha-Thalassemia

The decrease in *α*-globin chains in SCD reduces the mean corpuscular hemoglobin concentration and, thereby, the polymerization within the RBC. Homozygous alpha-thalassemia decreases the oxidative stress in SCA and could be an explanation for the protected role of alpha-thalassemia from various hemolysis-related complications of SCA. In contrast, the rate of vasoocclusive events is not related to their oxidative stress level [33]. In 2014, Cox et al. reported in a study of 601 stroke-free Tanzanian SCA patients aged <24 years, the importance of alpha-thalassemia of reducing risk of abnormal cerebral blood flow velocity [34]. Olatunya et al. performed a cross-sectional retrospective study on 100 young Nigerian patients with SCA and 63 controls. Their study confirms that coexistence of alpha-thalassemia with SCA significantly influences both the clinical and laboratory manifestations with increased bone pain crisis and protection against leg ulcers [35].

### 3.7. Fetal Hemoglobin Concentration

The Xmn1 polymorphism on chromosome 11p is the first genetic variant associated with increased levels of HbF [36], but it is assumed that additional factors are also necessary to have a clinical impact [27]. Another gene linked to higher HbF levels is BCL11* A* on chromosome 2p16. This is a transcriptional repressor of the *γ*-globin in helping control the developmental switch from embryonic to adult *ß*-globin [37]. Variations in the BCL11* A* binding site are found concurrent with HPFH [27]. The MYB transcription factor of the c*-*Myb gene on chromosome 6q23 is an important regulator of hematopoiesis, erythropoiesis, and HbF levels [27, 38]. Low MYB levels stimulate the erythroid differentiation, causing the release of larger cells expressing predominantly *γ*-globin [39]. Using whole genome sequencing of many SCD genomes, many other HbF regulators will be identified. With all the acquired new data, it will be difficult to define and harmonize the variable phenotypes [27].

### 3.8. G6PD

There are conflicting data on the impact of concurrent G6PD deficiency on laboratory and clinical parameters of SCD. Belisario et al. investigated a cohort of 395 Brazilian children with SCA and demonstrated that G6PD molecular deficiency or enzyme activity was not associated either with clinical ischemic stroke or high-risk transcranial Doppler [40], while Joly et al. studied 121 children with SCA and concluded that G6PD deficiency and absence of *a*-thalassemia increases the risk for cerebral vasculopathy in children with sickle cell anemia [41]. Antwi-Baffour et al. examined the blood of 120 SCD patients of genotypes HbSS and HbSC and found statistically significant differences between the Hb concentration of the participants having a G6PD deficiency (males) and those with normal G6PD activity (females), concluding that G6PD deficiency may increase the severity of anemia in SCD patients and therefore a possible earlier presentation [42].

## 4. Conclusion

SCD is a well-studied but not completely understood entity. When no newborn screening has taken place, a child's sickle cell disease can present for the first time in different ways and at different ages. Many factors are responsible for this. Familiarity and knowledge of SCD are of course essential with a low threshold for additional research (peripheral blood smear and Hb electrophoresis) to prevent delay of the diagnosis and performance of unnecessary tests.

## Figures and Tables

**Table 1:** Factors influencing the age of presentation of SCD in children.

Factors influencing the age of presentation of SCD in children
Doctor's delay
Patient's delay (including education and financial position of the parents)
High- versus low-resource country
Fetal hemoglobin
Reticulocyte count
Genetic modulators:
SCD genotype
Alpha-thalassemia
Fetal hemoglobin concentration
G6PD deficiency

## Data Availability

The data used to support the findings of this study are available in the archives of the ZNA Paola Children's Hospital, Antwerp, Belgium.
